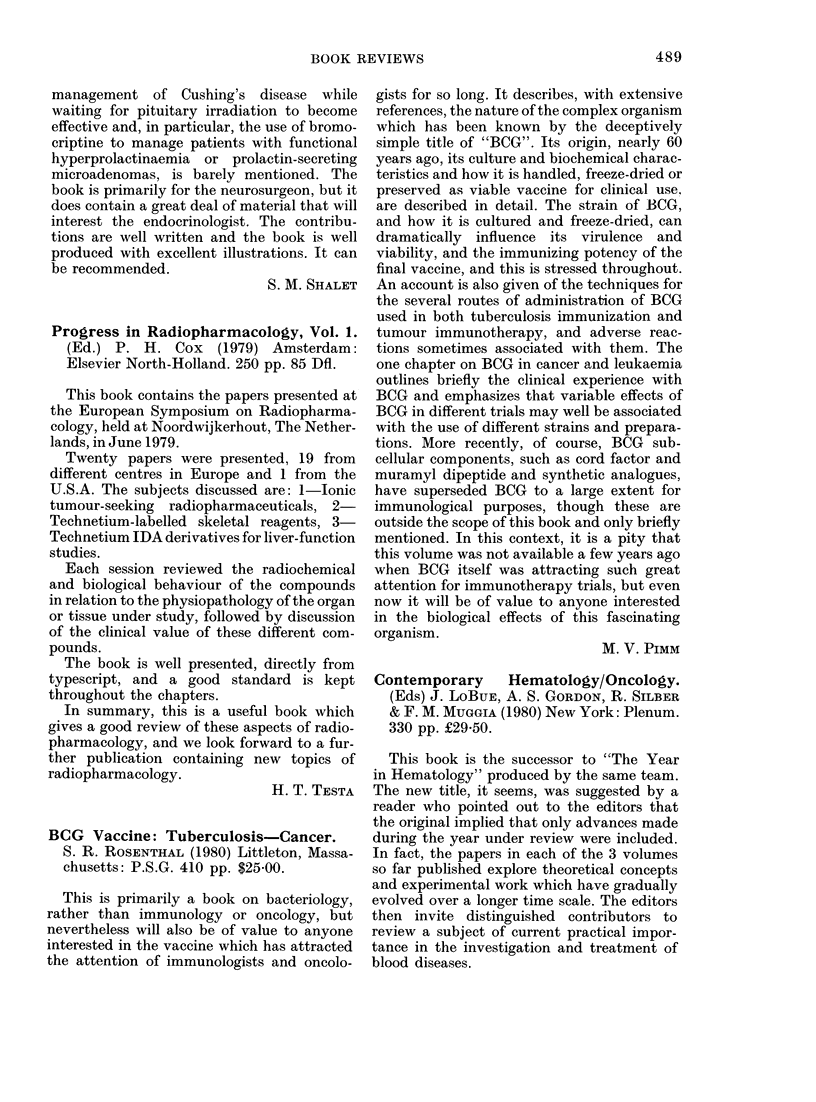# Progress in Radiopharmacology, Vol. 1

**Published:** 1980-09

**Authors:** H. T. Testa


**Progress in Radiopharmacology, Vol. 1.** (Ed.) P. H. COX (1979) Amsterdam: Elsevier North-Holland. 250 pp. 85 Dfl.

This book contains the papers presented at the European Symposium on Radiopharmacology, held at Noordwijkerhout, The Netherlands, in June 1979.

Twenty papers were presented, 19 from different centres in Europe and 1 from the U.S.A. The subjects discussed are: 1—Ionic tumour-seeking radiopharmaceuticals, 2—Technetium-labelled skeletal reagents, 3—Technetium IDA derivatives for liver-function studies.

Each session reviewed the radiochemical and biological behaviour of the compounds in relation to the physiopathology of the organ or tissue under study, followed by discussion of the clinical value of these different compounds.

The book is well presented, directly from typescript, and a good standard is kept throughout the chapters.

In summary, this is a useful book which gives a good review of these aspects of radiopharmacology, and we look forward to a further publication containing new topics of radiopharmacology.

H. T. TESTA